# Sacral Neuromodulator Surgery: Is There an Ideal Candidate?

**DOI:** 10.7759/cureus.93256

**Published:** 2025-09-26

**Authors:** Melissa Atallah, Ariel Zisman, Etan Eigner, Nicola Fazaa, Ameer Nsair, Valentine Shabataev

**Affiliations:** 1 Urology, Rambam Medical Center, Haifa, ISR

**Keywords:** over active bladder, patient’s satisfaction, sacral neuromodulation, under active bladder, voiding dysfunction

## Abstract

Objectives

Sacral neuromodulator implantation (SNM) is an advanced line of treatment for patients with overactive bladder (OAB) or underactive bladder (UAB). The procedure has two steps; patients who respond to temporary SNM proceed to permanent implantation. In this study, we aimed to evaluate the overall effectiveness of SNM in our patient population, as well as to identify potential correlations between patient demographics, clinical characteristics, and procedural outcomes. Additionally, we examined factors that may influence patient-reported satisfaction following SNM implantation.

Methods

A retrospective analysis was performed at the Department of Urology, Rambam Health Care Campus, Haifa, Israel, on individuals who underwent sacral neuromodulation between 2019 and 2024. Data collected included patient demographics, intraoperative variables, and procedural success rates. Baseline symptoms were compared to post-implantation symptoms in the OAB and UAB groups.

Results

A total of 47 patients underwent SNM implantation - 39 (83%) with UAB and eight (17%) with OAB. Of these, 79% proceeded to permanent SNM (implantation rate), and 68% retained the permanent device (success rate). Among the 25 with permanent SNM, 22 (88%) were satisfied, with an average reported satisfaction of 71%. In the UAB group, catheterizations decreased from four to two per day and pad use from six to two per day. In the OAB group, urinary frequency dropped from 16 to nine, nocturia from four to two, and pad use from 11 to three per day. No patient characteristics predicted success. Complications (local pain/infection) led to SNM removal in four (9%) patients.

Conclusions

SNM appears to be an effective treatment for both OAB and UAB patients, with significant reductions in catheterization and pad use, significant improvements in quality of life, and high self-reported satisfaction rates. Most removals occurred after the first stage, primarily due to lack of efficacy. Our findings suggest that intraoperative parameters and patient demographics have limited predictive value for success, emphasizing the need for improved selection criteria and individualized patient counseling prior to implantation, in order to optimize outcomes and minimize unnecessary procedures.

## Introduction

The lower urinary tract's main function is to store the urine and empty it. This function is regulated by a complex neural network located at various levels of the peripheral and central nervous system, which coordinates the activity of the bladder and the urethral sphincter [1]. An injury at different levels of this path may cause what was previously called a "neurogenic bladder". Nowadays, the correct term is neurogenic lower urinary tract dysfunction (NLUTD), which refers to abnormal function of either the bladder, bladder neck, and/or its sphincters related to a neurologic disorder [2]. Patients may suffer from overactive bladder (OAB), underactive bladder (UAB), or detrusor-sphincter dyssynergia (DSD), but non-urinary conditions such as sexual dysfunction, infertility, and bowel dysfunction are also common in patients with NLUTD [1,2].

Lower urinary tract symptoms (LUTS) encompass storage, voiding, and post-micturition symptoms, significantly impacting patients' quality of life [3]. Non-neurogenic lower urinary tract dysfunction refers to LUTS not attributed to neurological disorders. These dysfunctions can result from various factors such as metabolic syndrome, affective disorders, sex hormone deficiency, urinary microbiota, gastrointestinal functional disorders, and subclinical autonomic nervous system dysfunction [4]. LUTS are very common in the population, especially among women, with some studies reporting a prevalence of up to 79%, significantly impacting daily life [5].

Most patients initially undergo conservative treatment, including pelvic floor physiotherapy, self-catheterization, or pharmacological therapy, depending on the predominant component of their condition. However, a significant proportion fail to achieve adequate symptom relief, and these chronic conditions remain a therapeutic challenge [6]. For patients in whom conservative treatment is unsuccessful or unsuitable, sacral neuromodulation (SNM) with a sacral pacemaker is a viable option [7,8].

SNM is a minimally invasive technique that involves electrical stimulation of a sacral spinal nerve root to modulate a neural pathway in order to treat bladder or bowel disorders. The procedure consists of two steps: the temporary device is implanted first, and after a test period, the permanent one is installed. It's an accepted therapy for refractory urinary urgency and frequency, urgency urinary incontinence, non-obstructive urinary retention, and fecal incontinence [8,9]. Success rates range from 50% to 84%, depending on the underlying condition, according to a meta-analysis conducted in 2021 [10].

The current guidelines of the European Association of Urology (EAU) state that the SNM is an effective and safe option in the treatment of selective neurogenic LUTD, but lacks concrete guidance or specific recommendations. The ideal patient for SNM remains unclear due to the paucity of disease-specific studies [7].

This study aims to evaluate the efficacy of SNM surgery in reducing clinical symptoms in patients with OAB or UAB. We also examined the relationship between the procedural success and patient characteristics, including age, sex, Body Mass Index (BMI), underlying diseases, and the procedure's indication.

## Materials and methods

This retrospective cohort study was conducted by collecting data from medical folders of patients who underwent SNM implantation between the years 2019 and 2024.

All patients were under clinical follow-up at our institution, and the data collected included: gender, age, BMI, medical history including possible causes for NLUDT (diabetes, multiple sclerosis, spinal conditions), history of recurrent urinary tract infections, prior treatments, indication for SNM implantation, and time difference between diagnosis and SNM implantation. We also collected data on the surgical parameters during implantation (voltage needed for response, bellow response, and toe flex response), the need for procedural revision, causes for SNM implant removal, and complications (surgical infection, local pain, radiating leg pain).

We divided the patients into two groups: patients with UAB and patients with OAB. For the UAB group, we collected the number of catheterizations and pads used before and after the SNM implantation, and for the OAB group, the frequency, nocturia, and pads used before and after.

The SNM implantation was offered according to the current guidelines and only after failure of conservative treatment. All patients underwent transplantation of the Medtronic InterStimTM II system in a two-step procedure. In the first stage, the tined lead was implanted and connected to a temporary pulse generator, and in the second stage, the implantable pulse generator (IPG) was implanted. Only patients demonstrating at least 50% improvement in symptoms during the two-week test phase could proceed to the second stage. In patients who have not benefited from the SNM, the temporary implant was removed, and they were considered failed patients. 

After receiving the permanent SNM implant, patients were evaluated after four-six weeks, followed by assessments at three months, six months, and then annually.

During follow-up, patients were asked if they were satisfied with the SNM implant with a YES or NO response. If they answer YES, they will then rate their satisfaction on a scale from 0 to 100%.

Statistical analysis

SPSS version 28 (IBM Corp., Armonk, NY, USA) was used for all statistical analyses. P<0.05 was considered significant. Pearson chi-square tests were used for categorical parameters, and ANOVA or Kruskal-Wallis tests for continuous parameters. Differences between low and high satisfaction were tested with T-tests and Fisher's exact tests. Wilcoxon signed-rank tests were used to compare baseline symptoms to final symptoms.

## Results

A total of 47 patients underwent SNM transplantation between 2019-2024. 15 (32%) of them were males and 32 (68%) were females, with a mean age of 48.2 years. The implantation rate, defined as the proportion of patients who proceeded to permanent SNM implantation after the test phase, was 79%. The overall success rate, measured as the percentage of patients who retained the SNM without removal, was 68%. Demographic and clinical characteristics, including age, BMI, and medical history, did not significantly differ between patients who retained the implant and those who had it removed (Table 1).

**Table 1:** Patients' characteristics P-values were calculated using Pearson chi-square tests for categorical variables and ANOVA or Kruskal-Wallis tests for continuous variables, as appropriate. BMI: Body Mass Index; MS: Multiple Sclerosis; UTI: Urinary Tract Infection; OAB: Overactive Bladder; UAB: Underactive Bladder; CIC: Clean Intermittent Catheterization; SPT: Suprapubic Tube; TVT: Transvaginal Tape; SSI: Surgical Site Infection; SNM: Sacral Neuromodulator

Category/ Parameter		Removal after 1^st^ step (N=10)	Removal after 2^nd^ step (N=12)	Continued SNM Use (N=25)	All patients (N=47)	P-value
Gender	Male	5 (50%)	5 (42%)	5 (20%)	15 (32%)	P=0.24
	Female	5 (50%)	7 (58%)	20 (80%)	32 (68%)	
Age (years)		45.7±12.6	43.3±19.4	51.5±1307	48.2±15.2	P=0.26
BMI		30.1±5.3	26.4±4.3	30.0±5.3	29.1±5.2	P=0.11
medical history	Spinal conditions	5 (50%)	7 (58%)	16 (64%)	28 (60%)	P=0.74
	MS	2 (20%)	1 (8%)	1 (4%)	4 (8.5%)	P=0.31
	Diabetes	0	1 (8%)	4 (16%)	5 (11%)	P=0.36
	Fibromyalgia	0	1 (8%)	5 (20%)	6 (13%)	P=0.24
	Recurrent UTI	2 (20%)	4 (33%)	5 )20%)	11 (23%)	P=0.64
Indication	OAB	2 (20%)	0	6 (24%)	8 (17%)	P=0.18
	UAB	8 (80%)	12 (100%)	19 (76%)	39 (83%)	
prior treatments	Number	N=10	N=12	N=23	N=45	P=0.66
	Medications	3 (30%)	3 (25%)	9 (39%)	15 (33%)	
	CIC	4 (40%)	7 (58%)	9 (39%)	20 (44%)	
	Medications+CIC	3 (30%)	1 (8%)	3 (13%)	7 (16%)	
	SPT	0	0	1 (4%)	1 (2%)	
	TVT	0	0	1 (4%)	1 (2%)	
	SNM	0	1 (8%)	0	1 (2%)	
Time difference between diagnosis to SNM (years)		3 [1.8-4.8]	2 [2-5]	4.5 [1.6-6]	4 [2-6]	P=0.39
Surgical parameters	Bellow response	3.50±0.71	3.22±0.83	3.54±0.98	3.47±0.88	P=0.66
	Toe flex response	3.50±0.71	2.30±1.7	2.88±1.6	2.89±1.6	p=0.22
	Voltage	1.1±0.65	1.58±0.49	1.22±0.57	1.28±0.57	P=0.33
Need for revision		0	5 (42%)	3 (12%)	8 (17%)	P=0.022
Complications	SSI	0	4 (33%)	1 (4%)	4 (8.5%)	P=0.056
	Local pain	0	7 (58%)	6 (24%)	13 (28%)	P=0.008
	Radiating leg pain	0	3 (25%)	2 (8%)	5 (10.6%)	P=0.14

The most common reason for SNM removal was due to failure in 68% of patients (p <0.00005) (Table 2). 

**Table 2:** Reason for SNM removal P-value calculated using Pearson chi-square test.

Reason for SNM removal	Removal after 1^st^ step (N=10)	Removal after 2^nd^ step (N=12)	All patients (N=22)	P-value
Failure	10 (100%)	5 (42%)	15 (68%)	P= 0.036
Pain	0	1 (8%)	1 (5%)	
Failure+Pain	0	2 (17%)	2 (9%)	
Infection	0	4 (33%)	4 (18%)	

In total, 39 of the patients (83%) had UAB and eight (17%) had OAB. At the end of the follow-up period, 25 patients retained the SNM implant. Among them, six (24%) had OAB, while 19 (76%) had UAB. The follow-up period in those patients was 17.36 ± 11.7 months, with a minimal follow-up period of six months and a maximal follow-up period of 44 months. Among the 25 patients who retained the permanent SNM implant, 22 (88%) reported being satisfied with its function. The mean reported satisfaction level, assessed verbally on a scale from 0% to 100%, was 71%.

Following SNM implantation, significant symptom improvement was observed in both UAB and OAB patients (Table 3). In the UAB group, the number of catheterizations decreased from four to two per day (p=0.02), and the number of pads decreased from six to two per day (p=0.017). In the OAB group, urinary frequency decreased from 16 to nine (p=0.18), nocturia episodes decreased from four to two (p=0.058), and the number of pads used decreased from 11 to three per day (p=0.028).

**Table 3:** The effects of sacral neuromodulator (SNM) after implantation P-values were calculated using the Wilcoxon signed-rank test. “#” denotes “number” (e.g., number of catheterizations).

Group (N)	Symptom	Baseline	Final	P-value
Underactive bladder (UAB) (19)	Catheterization (#)	3.83±2.2	1.83±2.04	P=0.02
	Pads used (#)	6.20±5.7	2.2±2.0	P=0.017
Overactive bladder (OAB) (6)	Frequency (#)	16.2±7.8	9.4±3.9	P=0.18
	Nocturia (#)	4.0±1.4	2.0±1.8	P=0.058
	Pads used (#)	10.7±9.7	2.83±2.40	P=0.028

In the subgroup of patients with continued SNM use, satisfaction levels (above and below 50%) were analyzed in relation to demographic and surgical parameters. No significant correlation was found between any specific demographic factor and procedural success, nor with any intraoperative finding such as the voltage required for response or the number of leads for bellow or toe flex response (Table 4).

**Table 4:** Association between patient satisfaction and demographic/surgical parameters. P-values were calculated using the Pearson chi-square test for categorical variables, and the T-test or Mann-Whitney U test for continuous variables, as appropriate. MS: Multiple Sclerosis; SNM: Sacral Neuromodulator

Demographic/Surgical Parameters			Below<= 50%; n=8	Above 50%; n=17	P-value
Age			54.8±10.6	49.9±14.9	P=0.41
BMI			29.0±5.2	30.5±5.5	P=0.52
Gender	Male		0	5 (29%)	P=0.086
	Female		8 (100%)	12 (71%)	
medical history	Spinal conditions		4 (50%)	12 (71%)	P=0.39
	MS		1 (12.5%)	0	P=0.32
	Diabetes		2 (25%)	2 (12%)	P=0.57
	Fibromyalgia		2 (25%)	3 (17.6%)	P=1.00
Time difference between diagnosis to SNM (years)			5.5 [2.5-7.5]	4 [1-6]	P=0.32
Surgical parameters	Bellow response leads	0	0	1 (6%)	P=0.26
		2	0	2 (12.5%)	
		3	0	3 (19%)	
		4	8 (100%)	10 (62.5%)	
	Toe flex response leads	0	3 (37.5%)	2 (12.5%)	P=0.25
		2	0	2 (12.5%)	
		3	0	3 (19%)	
		4	5 (62.5%)	9 (56%)	
	Voltage		1.32±0.77; n=5	1.18±0.49; n=10	P=0.86

Additionally, in the subgroup of patients diagnosed with UAB, where a significant reduction in catheterization frequency was observed, no association was found between the reduction in catheterizations and any particular demographic characteristic.

In terms of complications, 13 patients (28%) experienced local pain, and five patients (11%) experienced a local infection, leading to SNM removal in four of them (9%). The main reason for the SNM removal after the second stage was failure in five out of 12 (46%) patients.

## Discussion

First described in the early 20th century, the SNM implant has become a promising solution for several urinary diseases [11].

SNM has been utilized in the treatment of detrusor overactivity, non-obstructive urinary retention, detrusor sphincter dyssynergia, and fecal incontinence due to incomplete spinal cord injury [8]. According to the EAU guidelines on neuro-urology from 2024, the SNM is an effective and safe option in the treatment of selective NLUTD patients, but there are no standardized criteria in terms of patient selection [7].

According to a systematic review and meta-analysis on patients with neurogenic lower urinary tract dysfunction undergoing SNM implantation, which included a total of 887 patients, the implantation rate was 66.2% [10]. Another study with 357 patients between the years 2000 and 2021 reported implantation rates of 58.8% [12]. In comparison, our study demonstrated a higher implantation rate of 79%. Notably, among patients with a permanent implant, 88% reported satisfaction with the therapy, emphasizing the positive impact of SNM on quality of life. Similarly, in a prospective, multicenter study with a five-year follow-up of OAB patients treated with SNM, Subjects showed improvement in all ICIQ-OABqol (International Consultation on Incontinence Modular Questionnaire) measures (p <0.0001) [13].

Additionally, in our study, UAB patients demonstrated a significant reduction in the number of daily catheterizations and pad usage, while OAB patients experienced decreased urinary frequency, nocturia, and pad use. These improvements are consistent with prior research that has highlighted SNM's role in reducing symptom burden in both conditions. In the previously mentioned prospective, multicenter study of patients with OAB treated with SNM, therapeutic success rates reached 82% [13]. Success was defined as a 50% or greater reduction in average daily leaks or voids due to urge incontinence or urgency-frequency, or a return to normal voiding patterns.

Zhang et al. performed a five-year retrospective, multicenter review of 247 patients who underwent SNM implantation. 47 (19%) patients had idiopathic urinary retention, 107 (44.1%) had neurogenic bladder, and 34 (13.7) had OAB. SNM appears effective in the long term with efficiency rates of 51.6%, 58.8% and 42.5% respectively [14].

According to a multicenter, retrospective case series on SNM in patients with detrusor underactivity, 77% demonstrated a favorable response to SNM, and 46.6% had a successful outcome by the end of the study. No significant difference was observed between men and women in success rates [15].

A multicenter study assessing predictors of success of SNM in patients with nonobstructive urinary retention (NOUR) demonstrated that 61% (46/76) of patients no longer required clean intermittent catheterization (CIC), and 88% (67/76) did not require additional treatment for NOUR after SNM implantation. This study also found that in women, younger age and psychiatric comorbidities (e.g., post-traumatic stress disorder (PTSD)) were predictive of first-stage success. Among men, factors such as younger age and a history of prior prostate or bladder neck surgery were similarly significant predictors of success [16].

In our study, we observed no statistically significant correlation between patient satisfaction and demographic factors such as age or gender, suggesting that SNM effectiveness is not influenced by these characteristics. Furthermore, when evaluating demographic correlations, no specific characteristic was found to be significantly associated with the reduction in catheterization frequency among UAB patients. This finding suggests that SNM efficacy in reducing the need for catheterization is independent of patient demographics. Those findings align with previous research indicating that age and gender do not significantly impact SNM outcomes. For instance, Banakhar and Hassouna found that patient satisfaction with SNM therapy was not correlated with age, duration of therapy, or complication rates [17]. Similarly, Meng et al. showed that SNM success is unrelated to age, and age alone should not be considered a limiting factor in SNM [18].

In contrast, Nasri et al. aimed to seek predictive factors and develop a predictive tool for SNM implantation. Four predictive factors were found, including age (<52y), gender (female), maximal urethral closure pressure (≥ 70 cmH2O), and the absence of an underlying neurological disease affecting the lower motor neuron [12]. These findings suggest that the literature is inconsistent, with conflicting studies regarding the predictors of procedural success. This discrepancy highlights the need for further research to better define predictive factors for SNM outcomes.

When assessing satisfaction levels (above and below 50%) in relation to intraoperative parameters in our study, no significant correlation was found between any specific parameter and procedural success. Similarly, a randomized prospective multicenter trial of 161 women undergoing first-stage SNM implantation reported no clear association between procedural success and intraoperative indicators, such as the number of electrodes that generated motor or sensory responses intraoperatively, the mean amplitude observed at responsive electrodes, or the lowest amplitude required to elicit a response. However, that study did find an association between a good amplitude response for bellows at electrode three and failure in the first stage, as well as a reduced improvement in daily urgency urinary incontinence episodes during that stage. Furthermore, after two years of follow-up, patients who exhibited an intraoperative sensory response at electrode three experienced a lower average reduction in daily urgency urinary incontinence episodes compared to those without any response [19].

In terms of adverse effects, in our study, 28% of patients experienced local pain, 11% developed a local infection, which led to SNM removal in four cases (9%). The primary reason for SNM removal after the second stage was treatment failure, occurring in five out of 12 patients (46%). These findings are consistent with previous reports on SNM-related complications. A systematic review and meta-analysis evaluating SNM in patients with NLUTD identified the most frequently reported adverse events as loss of treatment efficacy (4.7%), implant site pain (3.2%), lead migration (3.2%), and infection (3.6%) [10]. Another literature review from 2019 found that the most frequent complication with sacral neuromodulation is pain at the implant site (15%-42%), followed by lead migration (4%-21%), pain at the lead site (5.4%-19.1%), leg pain (18%), and infection (5.7%-6.1%) [20].

While the overall complication rates in our study appear higher, this may be attributed to differences in patient selection, follow-up duration, and reporting methodologies.

Despite the valuable insights gained from this study, several limitations should be acknowledged. First, the relatively small sample size, particularly in the OAB subgroup, may have limited the statistical power to detect certain differences. Additionally, patient satisfaction was assessed subjectively through verbal reports rather than with standardized questionnaires or objective measures such as follow-up urodynamic testing. While this limits the ability to precisely quantify functional improvements, it reflects real-world patient experiences and perceived benefits from SNM therapy. Importantly, despite these limitations, our study demonstrates the clinical utility of SNM in daily practice, highlighting its potential to improve symptoms and quality of life in patients with UAB and OAB. Future prospective studies with larger cohorts and objective outcome measures will further validate these findings and optimize patient selection criteria for SNM.

## Conclusions

In this study, we evaluated clinical and intraoperative factors associated with SNM outcomes among patients with OAB and UAB. We found that SNM led to meaningful improvements in symptoms, particularly reduced catheterization and pad use, especially in the UAB group, and was associated with significant improvements in quality of life and high self-reported satisfaction rates.

Despite these encouraging outcomes, a significant proportion of removals occurred following the first stage, predominantly due to a lack of treatment efficacy. Notably, intraoperative parameters such as bellow and toe responses, as well as patient demographic or clinical characteristics, did not consistently predict success. These findings highlight the ongoing challenge of identifying optimal candidates for SNM therapy. The limited predictive value of current intraoperative and baseline variables emphasizes the importance of refining selection criteria. Future studies should focus on developing robust preoperative assessment tools and exploring potential parameters that may better forecast treatment outcomes. Improved patient selection may lead to better clinical outcomes, fewer unnecessary procedures, and more cost-effective use of SNM in clinical practice.
